# Acute Aortic Injury and Bilateral Pulmonary Emboli in the Setting of COVID-19

**DOI:** 10.7759/cureus.38330

**Published:** 2023-04-30

**Authors:** Christopher C Garnett, Austin Poist, Chase Lazenby, Jeffrey Nielson, Rebecca Perry

**Affiliations:** 1 Emergency Medicine, Kettering Health Network, Dayton, USA

**Keywords:** post-acute covid-19 syndrome, covid-19, aortic dissection, pulmonary embolus, aortic injury

## Abstract

In this report, we present the case of a 72-year-old female diagnosed with an aortic dissection variant and bilateral pulmonary emboli (PE) in the setting of Coronavirus Disease of 2019 (COVID-19) infection. The patient was transported from home to the emergency department (ED) via emergency medical services (EMS) with acute chest pain and dyspnea. After arriving at the ED, she was hypoxic on her baseline supplemental O2 requirement and tachycardic and tachypneic. Computed tomography (CT) angiogram of the chest showed evidence of possible thoracic aortic dissection and bilateral PE. The patient was ultimately transported to a tertiary center for operative aortic repair and bilateral embolectomy and, fortunately, survived the procedures. Interestingly, during operative repair of the aorta, no obvious dissection flap was noted, but rather evidence of a limited tear in the intimal layer of the aorta. This is an interesting case as acute aortic injuries in the setting of COVID-19 infection have not been as widely documented as PE in the setting of COVID and highlight the need for further research on the possible association between them.

## Introduction

The increased incidence of deep venous thrombosis (DVT) and pulmonary emboli (PE) in the setting of COVID has been well documented, with some reports showing up to 14.8% and 16.5%, respectively [1]. However, acute aortic dissection in the setting of COVID has not been as widely noted. Several case reports have discussed the incidence of acute aortic pathology with COVID infection [2,3], and this case is another example of such. The case presented continues to point towards the need for increased research on the possible link between acute aortic injuries and COVID.

This case report was originally presented at the Dayton Area Graduate Medical Education Community (DAGMEC) Annual Virginia C. Wood Resident Research Forum in April 2022.

## Case presentation

The patient is a 72-year-old female who tested positive for COVID-19 22 days before presenting to the ED. She had two short hospitalizations when she was initially diagnosed with COVID. Due to dyspnea and hypoxia, she was ultimately discharged home on 2 L of supplemental oxygen after her first hospitalizations.

On this day, she presented to the ED again with new chest pain and worsening shortness of breath. The patient described having intermittent midsternal chest pressure with exertion that started seven hours before arrival in the ED and two days of gradually worsening shortness of breath. Her initial peripheral capillary oxygen saturation (SpO2) was 89% on 2 L of oxygen, which improved with increasing oxygen to 4 L. The patient was initially tachycardic and tachypneic.

**Figure 1:**
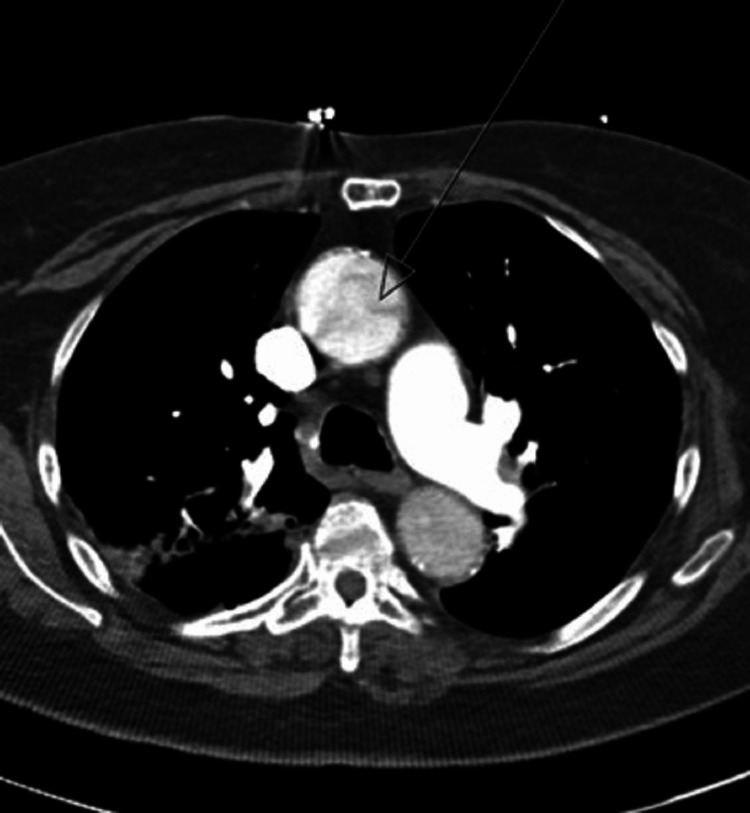
CT angiogram of thoracic aorta concerning for dissection.

**Figure 2:**
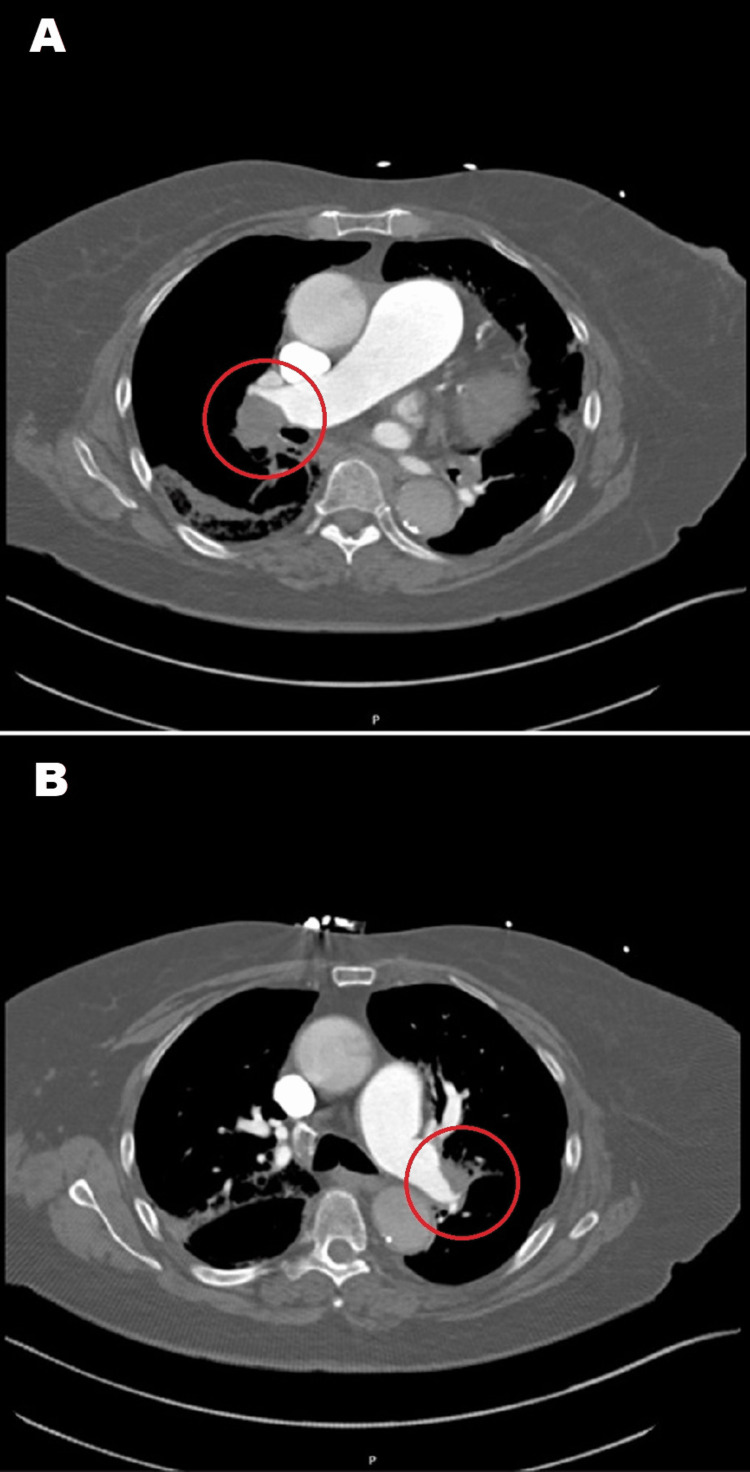
A) Right pulmonary embolus. B) Left pulmonary embolus.

She was initially empirically treated with aspirin for possible acute coronary syndrome and antibiotics for possible secondary bacterial pneumonia before obtaining a chest CT angiogram. The CT angiogram showed evidence of bilateral PE, as well as evidence of a type A aortic dissection. Figure 1 shows the CT angiogram findings concerning aortic dissection, and Figure 2 shows evidence of bilateral PE.

Esmolol infusion was promptly started, and cardiothoracic surgery was consulted, who recommended transfer to a tertiary center for dissection repair and thrombectomy. No anticoagulation was started in the ED per cardiothoracic surgery’s recommendation to proceed with surgical intervention. During the procedure, no obvious dissection flap was found. However, a transverse intimal tear in the proximal descending aorta was recognized, and a hemiarch replacement and bilateral thrombectomy were performed. The patient was discharged in stable condition on postoperative day eight.

## Discussion

Aortic dissection is the result of rupturing of the intimal layer of the aortic wall and separation of the intima-media space, creating a false lumen for blood to enter and further propagate separation [4]. The initiating event is typically unknown; however, it is usually thought to occur from a structural abnormality in the aortic wall and/or systemic hypertension [4]. Aortic dissection is most frequently diagnosed by CT imaging, but mediastinal widening in plain chest radiography has been documented in acute aortic dissection in up to 37.4% of cases [4].

In this case, the patient had evidence of aortic dissection on CT angiogram. However, the surgeon’s operative note documented that there was not a frank dissection flap but evidence of an acute limited intimal tear (LIT). An acute LIT is a rare aortic lesion considered an aortic dissection variant [5]. It is an incomplete dissection or laceration of the intima and adjacent media without substantial blood dissection into the false lumen created [5]. As LITs in the thoracic aorta are considered a dissection variant, they are typically managed surgically [5], as was the case in the patient in this report.

Early detection of acute aortic injury, especially Type A aortic dissections, is vital due to the high morbidity and mortality associated with aortic dissection. Overall in-hospital mortality for all types of aortic dissection has been documented as high as 27.4%. Mortality has also increased in Type A dissections from 26% when managed surgically to 58% when surgery is not received [4]. Thus, early detection of aortic pathology in COVID patients with an increased mortality risk is necessary for optimal outcomes. Thankfully, findings concerning acute aortic injury were noted on this patient’s CT angiogram of the chest, which was optimized for PE diagnosis. However, there could be instances where subtle, early aortic injuries are missed in COVID patients if they are not considered in the ED patient with presumed PE.

As mentioned previously, aortic dissection in the setting of COVID has not been extensively documented. As such, little research has been published on the possible pathophysiologic links between the two. Some articles have postulated that similar molecular pathways are activated in patients with COVID-19 and patients with Marfan Syndrome and aortic dissection [2]. However, there is not currently enough evidence that COVID-19 has a similar pathway and, therefore, insufficient evidence to definitively say that COVID-19 correlates with aortic dissection [2].

## Conclusions

It has been documented that COVID infection can induce a thrombogenic state, placing patients at risk for developing pulmonary embolism. However, a less-known complication of COVID infection is aortic dissection. There have been postulated mechanisms linking the two, such as COVID-19 inducing a proinflammatory state in the setting of an individual with other risk factors for aortic dissection. As possible pathophysiologic responses predispose patients with COVID infection to aortic dissection, this case highlights the need for further research to determine if there is an increased risk of aortic dissection in patients with COVID infection.
